# Laparoscopic Resection of Recurrence from Hepatocellular Carcinoma after Liver Transplantation: Case Reports and Review of the Literature

**DOI:** 10.1155/2016/8946471

**Published:** 2016-03-01

**Authors:** Mushegh A. Sahakyan, Airazat M. Kazaryan, Ewa Pomianowska, Andreas Abildgaard, Pål-Dag Line, Bjørn Atle Bjørnbeth, Bjørn Edwin, Bård Ingvald Røsok

**Affiliations:** ^1^The Intervention Centre, Oslo University Hospital, Rikshospitalet, 0027 Oslo, Norway; ^2^Institute of Clinical Research, Medical Faculty, University of Oslo, 0318 Oslo, Norway; ^3^Department of Surgery No. 1, Yerevan State Medical University after M. Heratsi, 0025 Yerevan, Armenia; ^4^Department of Gastrointestinal Surgery, Akershus University Hospital, 1478 Lørenskog, Norway; ^5^Department of HPB Surgery, Oslo University Hospital, Rikshospitalet, 0407 Oslo, Norway; ^6^Department of Radiology, Oslo University Hospital, Rikshospitalet, 0027 Oslo, Norway; ^7^Department of Transplantation Medicine, Oslo University Hospital, Rikshospitalet, 0424 Oslo, Norway

## Abstract

*Background*. Recurrence of hepatocellular carcinoma (HCC) after liver transplantation (LT) indicates a poor prognosis. Surgery is considered the only curative option for selected patients with HCC recurrence following LT. Traditionally, the preference is given to the open approach.* Methods*. In this report, we present two cases of laparoscopic resections (LR) for recurrent HCC after LT, performed at Oslo University Hospital, Rikshospitalet.* Results*. Both procedures were executed without intraoperative and postoperative adverse events. Whereas one of the patients had a recurrence one year after LR, the other patient did not have any sign of disease during 3-year follow-up.* Conclusions*. We argue that, in selected cases, patients with HCC recurrence following LT may benefit from LR due to its limited tissue trauma and timely start of subsequent treatment if curative resection cannot be obtained. In patients with relatively favorable prognosis, LR facilitates postoperative recovery course and avoids unnecessary laparotomy.

## 1. Introduction

Hepatocellular carcinoma (HCC) is the fifth most common cancer and the second leading cause of cancer related death in the world with more than a half million new cases annually [1, 2].

The key aspect in successful treatment of HCC is linked to proper preoperative staging. The most widely adopted staging system is the Barcelona Clinic Liver Cancer (BCLC) system, based on tumor stage, severity of liver disease, and performance status of the patients to different therapeutic alternatives. According to BCLC recommendations, the patients with early HCC are suitable candidates for radical treatment (i.e., resection, liver transplantation, or radio frequency ablation). Among these approaches, liver transplantation (LT) seems to be an attractive therapeutic option, particularly in patients with significant liver parenchymal disease (Child-Pugh B and C), as it has the potential to cure both the tumor and underlying liver disease [3].

However LT is often limited to early stages of disease in order to ensure outcomes that are comparable to those of liver transplantation for noncancer related indications. Selection criteria, such as Milan (a single tumor ≤ 5 cm or ≤ 3 tumors ≤ 3 cm without signs of macrovascular invasion), UCSF (a single tumor ≤ 6.5 cm, or ≤ 3 tumors with the largest being ≤4.5 cm, and a total tumor burden of ≤8 cm), and up-to-seven (the sum of the number of nodules and of the diameter of the largest tumor (in cm) not exceeding seven, in absence of macrovascular invasion and extrahepatic spread), have been introduced up to date [4–8].

At the same time, recurrence of HCC after LT still occurs and is to a large extent related to tumor stage. Relapse after transplantation is usually associated with poor prognosis, since in most cases it must be considered as a sign of systemic disease. Extrahepatic lesions, present in majority of these patients, are not amenable to curative treatment, thereby being considered for palliative therapy only. However, the efficacy of the latter remains unsatisfactory [9]. Several authors emphasized the curative role of surgery for HCC recurrences, indicating that longer time interval between LT and the recurrence and feasibility of resection are associated with improved survival [10–12].

Today, laparoscopic surgery is comparable to open technique for primary resection in a large fraction of patients with HCC [13]. However, in terms of tumor recurrences, a conventional open technique is usually preferred, perhaps considering the fact that patients had previously undergone major surgical procedure. In our review of current literature, we are not aware of any report concerning laparoscopic approach for HCC recurrence after LT. Herein we report on two cases of recurrences following LT that were treated by laparoscopic resections at our institution.

## 2. Materials and Methods

Hospital records review revealed that 89 patients with HCC on final pathology underwent LT at Oslo University Hospital, Rikshospitalet, from 2004 to 2014. Eighteen of these patients (20.2%) had recurrence during follow-up. Of these, two with locoregional isolated recurrences and performance status of 0 according to Eastern Cooperative Oncology Group (ECOG) scale were considered eligible for curative resection. Laparoscopic approach was applied.

## 3. Results

### 3.1. Case 1

A 62-year-old male underwent LT for multifocal HCC, combined with hepatitis C related cirrhosis. According to preoperative CT scan images, a massive lesion with greatest extent over 8 cm was found in the right lobe and 2-3 small lesions were found in segment 2. AFP level was elevated at 278 ng/mL. Histological examination of the explanted liver revealed disseminated HCC with numerous nodules in all segments and widespread vessel infiltration. In the posttransplant period the patient received immunosuppressive therapy with tacrolimus, prednisolone, and mycophenolate mofetil (MMF).

**Figure 1:**
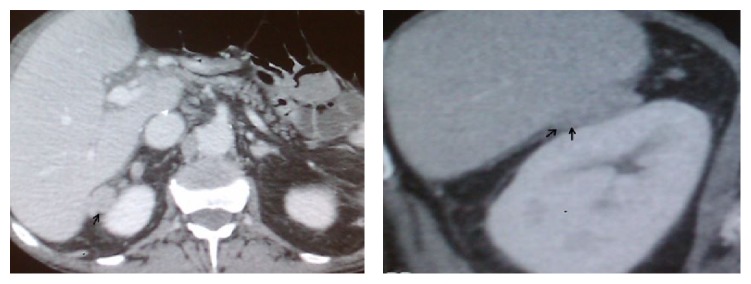
CT with intravenous contrast. Extrahepatic lesion between liver segment 6 and right kidney, largest diameter 2 cm.

Nine months after LT a routine CT scan revealed an extrahepatic, 2 cm lesion adjacent to segment 6 (Figure 1). Ultrasound guided biopsy verified HCC. The case was determined suitable for laparoscopic resection.

Pneumoperitoneum was established through an open approach via right pararectal region, supplemented by three trocars: a 12 mm through the scar from previous incision and two 5 mm in the right flank. After complete mobilization of the right lobe, the tumor was found retroperitoneally between the right kidney and adrenal gland. Intraoperative ultrasound was carried out to determine the borders of the tumor. Despite close relation with renal capsule, no signs of infiltration to the kidney were revealed. Division of Gerota's fascia and exposing the upper part of the kidney allowed mobilization of the tumor laterally and cranially, separating it from the renal capsule. No evidence of infiltration to adjacent structures was found and the tumor was removed. No significant bleeding occurred during the surgery and the operation time was 131 min.

Histological findings confirmed the presence of two (40 mm and 12 mm) extrahepatic recurrences from HCC. The postoperative course was uneventful.

Surveillance for the first 8 months revealed no signs of disease recurrence, but one year after the reresection, metastases in liver, lungs, and adrenal gland were detected. Subsequent therapy with sorafenib was considered as the only possible active treatment.

### 3.2. Case 2

HCC was diagnosed in a 57-year-old male with hepatitis C and cirrhosis. A 3 cm tumor, located in segment 5, and two small hypervascular lesions, suggestive of HCC, were discovered by CT scan. The AFP level was significantly elevated (>20.000).

The patient was assessed and accepted as a liver recipient and LT was performed. The postoperative course was uneventful and the patient received immunosuppression with tacrolimus, prednisolone, and MMF.

**Figure 2:**
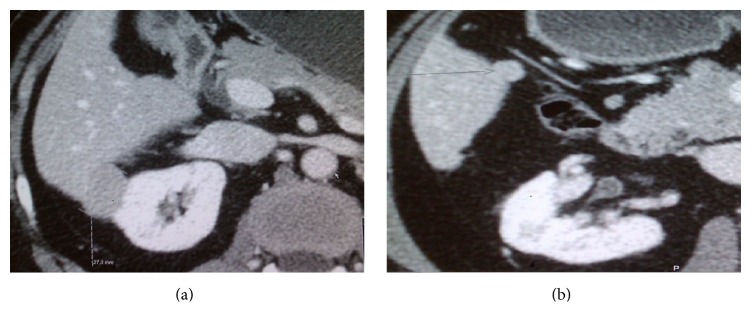
CT with intravenous contrast. Two lesions detected. First lesion adjacent to lateral part of right kidney, largest diameter 2.7 cm (a). Second lesion close to duodenum, largest diameter 1.4 cm (b).

An abdominal CT scan, 6 years after LT, detected two intrahepatic lesions. The larger one, measuring 2.7 cm, was located laterally in segment 6, close to the right kidney (Figure 2(a)). The second lesion of 1.4 cm was located between the segments 5 and 6, adjacent to the wall of duodenum (Figure 2(b)).

The laparoscopic approach was chosen for reresection. Pneumoperitoneum was obtained through an open access technique via umbilicus. Adhesiolysis was carried out. Afterwards, one 12 mm trocar was placed high in the midline, followed by two 5 mm trocars in the right pararectal area and more laterally. Mobilizing the right lobe of the liver, the lateral tumor was found to infiltrate into Gerota's fascia. Using LigaSure (Valleylab/Covidien, Boulder, CO, USA) device, the tumor was completely mobilized and separated from the fascia. Finally, both of the tumors were removed by wedge resection of the liver. Operative time was 127 min with only minor intraoperative blood loss observed.

A pathological examination revealed 32 mm and 18 mm measuring moderately differentiated HCC with trabecular pattern. The postoperative course was uneventful. During further surveillance for more than 3 years, no signs of disease recurrence have been observed.

## 4. Discussion

LT is associated with significantly higher 5-year overall and disease-free survival rates, compared to LR (65.7% versus 43.8%; *P* = 0.005 and 85.3% versus 22.7%; *P* < 0.001, resp.); hence it should be considered in patients with liver disease and cirrhosis within the various transplantation criteria [14, 15]. Unlike resection, LT is also an ultimate solution for treatment of HCC and underlying cirrhosis [16]. Up to 83% 5-year recurrence-free survival rates have been reported after LT for HCC, which is comparable to LT for nonmalignant disease [17].

Unfortunately, even the utilization of criteria for LT does not prevent tumor recurrence. According to Welker and coworkers [10], the recurrence rates following LT vary between 15% and 20%. The latter was assumed to be dependent from patient selection criteria for initial LT. In another paper by Sotiropoulos and colleagues [18] a 12.6% recurrence rate was found among the patients within the Milan criteria.

Numerous retrospective analyses have been performed to estimate the factors leading to the recurrence and eventual poor outcome. Parameters such as vascular invasion, poor differentiation, tumor size >5 cm, number of lesions, HCC exceeding the Milan criteria, and elevated pretransplant AFP levels have been reported to have significant impact on the rate of posttransplant recurrences [18–20].

The limitations of therapeutic options for recurrent HCC after LT lead to an absence of established treatment strategies [18, 21]. A major contraindication for reresection is the presence of extrahepatic recurrences that have been described in 10–43% of patients. These are typically located in lungs, bones, abdominal lymph nodes, and adrenal glands [22]. Nevertheless, even though disseminated disease commonly precludes curative treatment, patients with limited intra- or extrahepatic lesions may still benefit from local treatment, including surgical resection [17]. Roayaie et al. [12] reported that recurrent disease should be treated by surgery whenever possible, since it was independently associated with prolonged survival of 47% at 5 years. Hwang et al. [23] recommended performing pulmonary metastasectomy for resectable HCC metastases in lungs following LT, as it may provide a chance of long-term survival in a substantial proportion of patients. According to recent clinical studies, resection of both intra- and/or extrahepatic lesions in a selected group of patients can be associated with an improved overall 5-year survival ranging from 27 to 88% [22, 24–26]. Furthermore, the authors conclude that even noncurative surgery seems to prolong survival [18]. Reportedly, resection rates for recurrent HCC may vary from 31 to 43% among different centers [19]. However, the studies are limited by retrospective design and a small series of cases, reported so far (Table 1).

Another available surgical option seems to be retransplantation. Nevertheless, the attempts are currently made to salvage graft rather than performing retransplantation, because of the organ shortage. Hence, although the resections are relatively rare, they are associated with favorable outcome in selected patients and avoid retransplantation in most of the cases [27].

There has been a considerable development in laparoscopic liver surgery during the last decade and it is now generally accepted that laparoscopic resection offers advantages, compared to the open approach in terms of severity of complications, 30-day readmission rate, length of hospital stay, and blood loss [28]. However, no reports in the literature concerning laparoscopic resections for recurrent HCC after LT have been observed to date.

In this report we present two cases that may suggest that laparoscopic resection is a feasible and safe procedure in selected patients with recurrent HCC following LT. Since these patients often have a dismal prognosis, emphasis should be on minimizing surgical risk and trauma when choosing therapeutic options for treatment of recurrence. In this context laparoscopy seems to be a rational approach that may be well suited in selected patients. The disparity in long-term outcome in two patients can probably be related to the difference between the risk factors for recurrence. While the first patient exceeded all morphological criteria systems, the second patient fulfilled each of them (BCLC, Milan, UGSF, and “up-to-seven”). On the other hand, the occurrence of so-called “de novo” cancer cannot be excluded in Case 2, considering the fact that the lesions developed 6 years after LT.

In the light of the limited role of medical therapy in palliative treatment of recurrent HCC, patients with poor prognosis and limited survival prospects can benefit from laparoscopy due to shortened recovery period. In case of indications for subsequent therapy, laparoscopy may avoid potential delay in treatment, caused by open surgery. Conversely, the patients with favorable prognosis may also benefit from the fast recovery course, provided by laparoscopy. Extensive experience in the field of laparoscopic surgery coupled with the evaluation of therapeutic options will establish the new surgical guidelines in treatment of resectable recurrent HCC after LT.

## Figures and Tables

**Table 1:** Recurrence and resection rates following liver transplantation.

Authors	Year of publication	Liver transplantations	Total number of recurrences	Resections for recurrent HCC
Pfeiffenberger et al. [29]	2013	136	18	3 (16.6%)
Hwang et al. [23]	2012	587	43	23^*∗*^
Chok et al. [16]	2011	139	24	17 (70.8%)
Pfiffer et al. [24]	2011	139	24	7 (29.1%)
Kornberg et al. [17]	2010	60	16	7 (44%)
Shin et al. [30]	2010	138	28	4 (14.3%)
Valdivieso et al. [19]	2010	182	23	11 (47.8%)
Taketomi et al. [25]	2010	101	17	9 (52.9%)
Roayaie et al. [12]	2004	311	57	15 (32%)
Schlitt et al. [11]	1999	69	39	15 (38%)
Regalia et al. [26]	1998	132	21	7 (32%)

^*∗*^Hwang et al. reported on 43 HCC cases with pulmonary recurrences after LT. 23 patients underwent resections.
